# New Thermal and Microbial Resistant Metal-Containing Epoxy Polymers

**DOI:** 10.1155/2010/976901

**Published:** 2010-06-24

**Authors:** Tansir Ahamad, Saad M. Alshehri

**Affiliations:** Department of Chemistry, King Saud University, Riyadh11451, Saudi Arabia

## Abstract

A series of metal-containing epoxy polymers have been synthesized by the condensation of epichlorohydrin (1-chloro-2,3-epoxy propane) with Schiff base metal complexes in alkaline medium. Schiff base was initially prepared by the reaction of 2,6 dihydroxy 1-napthaldehyde and *o*-phenylenediamine in 1  :  2 molar ratio and then with metal acetate. All the synthesized compounds were characterized by elemental, spectral, and thermal analysis. The physicochemical properties, viz., epoxy value, hydroxyl content, and chlorine content [mol/100 g] were measured by standard procedures. The antimicrobial activities of these metal-containing epoxy polymers were carried out by using minimum inhibitory concentration (MIC) and minimum bactericidal concentration (MBC) methods against *S. aureus*, *B. subtilis* (Gram-positive bacteria), and *E. coli*, *P. aeruginosa* (Gram-negative bacteria). It was found that the ECu(II) showed higher antibacterial activity than other metal-chelated epoxy resin while EMn(II) exhibited reduced antibacterial activity against all bacteria.

## 1. Introduction

Since the last two decades, several thermal and microbial resistant polymers have been synthesized by the immobilization of metal complexes into the polymers [1] and used as a thermal resistant, microbial resistant, scratch resistant, and flame retardant-coating materials. Some metal complexes commonly used in the synthesis of metal containing polymers are Schiff base, ferrocene, Imidazole, secondary and tresiory amine metal complexes, and so forth, [2, 3]. Among these metal complexes Schiff base metal complexes have been widely used due to their corrosive resistant, microbial as well as thermal resistant properties [4]. Epoxy polymers are one of the most important higher-performance polymer systems in use today, ranging from simple two-part adhesives and sports equipment to high-tech applications such as formula one racing cars and the aerospace industry. Epoxy polymers are capable of undergoing homopolymerisation, although this process generally yields products with inadequate properties for high-tech applications. Consequently, in many cases catalysts, additives, and cocuring-agents are formulated with the epoxy resin to significantly increase the storage stability, decrease the cure time, and improve the final properties [5, 6]. The use of metals to formulate resin systems with excellent storage stability is discussed, along with the use of coordination compounds to improve cured resin properties such as fracture toughness, thermal stability, and water absorption [7, 8]. Two approaches are generally used for the attachment of metal complexes with polymers. The first approach involves the introduction of the bifunctional metal complexes as a monomer, followed by their polymerization [9]. The second approach involves the linking of metal complexes directly onto preformed functional polymers [10]. The first approach has the advantage that the monomer can be polymerized with several other comonomers, and the composition can be varied easily. These facts propagated our interest at this time to synthesize new materials with antimicrobial and thermal resistance properties. In the present study, Schiff base metal complexes were reacted with epichlorohydrin in 1.25  :  1 molar ratios to produce a series of E-M(II) metal containing epoxy polymers. The characterization of the new epoxy polymers was done with the purpose of proposing their structures and determining their specific applications as thermally resistant and/or microbial resistant materials.

## 2. Experimental

### 2.1. Materials and Reagents

o-phenylenediamine, epichloro-hydrin, manganese(II) acetate tetrahydrate [Mn(CH_3_COO)_2_-4H_2_O], copper(II) acetate monohydrate [Cu(CH_3_COO)_2_-H_2_O], nickel(II) acetate tetrahydrate [Ni(CH_3_COO)_2_-4H_2_O], cobalt(II) acetate tetrahydrate [Co(CH_3_COO)_2_-4H_2_O], and zinc(II) acetate dihydrate [Zn(CH_3_COO)_2_-2H_2_O] (Sigma Aldrich) were used without furtherpurification. The solvents, such as dimethylformamide (DMF), dimethyl sulfoxide (DMSO), ethyl alcohol, methan-ol, and acetone, were distilled before use. 2,6-hydroxy naphthaldehyde was prepared according to the literature [11].

### 2.2. Measurements

**Characterization for UV, FTIR, and NMR**

The epoxy value (mol/100 g) of resin was determined by analytical method [12]. This method was based on the back titration. 0.2 g of resin was added to 30 cm^3^ of 0.1 M-HCl and mixed for 2 h. Then unreacted HCl was retitrated with phenolphthalein by standard alkali solution using the following formula:

(1) $$\text{w}\left(\text{EP}\right)=\frac{\left(V_{2}-V_{1}\right)\cdot c\left(\text{NaOH}\right)\;\times \;0.043}{\text{mass}\;\text{of}\;\text{sample}\;},$$

where *V*_1_ is the volume of NaOH solution used for blank and *V*_2_ is the volume of NaOH solution used for sample.

Hydroxyl content was determined by acetylation with acetyl chloride in pyridine. The excess of acetyl chloride was decomposed with water and the resulting acetic acid, formed both in hydrolysis and in the acetylation process, was titrated with standard alkali using the following formula:

(2) $$\text{w}\left(\text{OH}\right)=\frac{\text{mass}\;\text{of}\;\text{sample}\;}{\left(V_{1}-V_{2}\right)\cdot \;c\left(\text{KOH}\right)\times 170\;},$$

where *V*_1_ is the volume of KOH solution used for blank and *V*_2_ is the volume of KOH solution used for sample. The chlorine content was determined by treating the resin solution with alcoholic KOH and titrating it against standard HCl [13]

(3) $$\left(\text{Cl}\right)=\frac{\text{c}\left(\text{KOH}\right)\cdot V\times 0.0355}{\text{mass}\;\text{of}\;\text{sample}\;}.$$

### 2.3. Synthesis of Schiff Base Ligand (H_2_L)

Schiff base ligand was prepared by (1.88 g, 0.01 mol) of monoaldehyde and was dissolved in 8 mL of THF, and the solution was added to a THF/ MeOH (1;1 10 mL) solution of o-phenylenediamine (0.9 g, 0.005 mol) and refluxed for 6 hours. With continuous stirring the color of the solution has been changed reddish. The progress of reaction was monitored by thin layer chromatography (TLC). The reaction mixture was cooled and precipitated into 20 mL MeOH. The reddish purple colour precipitate was filtered, then washed with methanol, and dried in vacuum, Yield 45% (2.01 g).

### 2.4. Synthesis of Schiff Base Metal Complexes

A solution of 2.13 g (0.1 mol) of H_2_L and 0.05 mol of metal acetate hydrates in 25 cm^3^ of ethanol were stirred for 2 h at 70°C. Then, the mixture was cooled, filtered, and washed with methanol to give the colored metal complexes, and the pure product was obtained after recrystallization from methanol. All the metal complexes used in this study have been characterised using similar methods. The colour, yield, and spectral and elemental data of the complexes are given next. 

MnL. Reddish purple, 3.64 g, 72% yield. FTIR [KBr pellets, *υ*(max), cm^−1^]: 3360, 3050,1640, 1530, 748, 620, 550, MALDI-TOF MS (m/z): 502.61 [M + H^+^], Anal. Calcd for C_28_H_18_O_4_N_2 _-Mn(II): C, 67.07%; H, 3.62%; N, 5.59%; Mn, 10.96%. Found: C, 67.08%; H, 3.63%; N, 5.57%; Mn, 10.94%.

CoL. Dark brown, 3.54 g, 70% yield. FTIR [KBr pellets, *υ*(max), cm^−1^]: 3360, 3050,1640, 1538, 748, 620, 540, MALDI-TOF MS (m/z): 506.03, Anal. Calcd for C_28_H_18_O_4_N_2 _-Mn(II): C, 66.54%; H, 3.59; N, 5.54%; Co, 11.66%. Found: C, 66.54%; H, 3.60%; N, 5.55%; Co, 11.68%.

NiL. Purple, 3.69 g, 73% yield. FTIR [KBr pellets, *υ*(max), cm^−1^]: 3360, 3055,1642, 1540, 745, 625, 540, MALDI-TOF MS (m/z): 505.12, Anal. Calcd for C_28_H_18_O_4_N_2_-Ni(II): C, 66.57%; H, 3.59; N, 5.55%; Co, 11.62%. Found: C, 66.58%; H, 3.61%; N, 5.56%; Ni, 11.63.

CuL. Dark purple, 3.52 g, 69% yield. FTIR [KBr pellets, *υ*(max), cm^−1^]: 3360, 3050,1645, 1535, 748, 620, 545, MALDI-TOF MS (m/z): 510.6, Anal. Calcd for C_28_H_18_O_4_N_2_-Cu(II): C, 65.94%; H, 3.56; N, 5.49%; Cu, 12.62%. Found: C, 65.95%; H, 3.57%; N, 5.51%; Co, 12.63%.

ZnL. Reddish purple, 3.62 g, 71% yield. ^1^H-NMR (300 MHz, DMSO, d): 9.56 (2H, OH), 7.52–6.45 (12 hours, Ar-H), 9.16 (2H, CH=N), FTIR [KBr pellets, *υ*(max), cm^−1^]: 3360, 3055,1645, 1540, 750, 620, 540, MALDI-TOF MS (m/z): 511.42, Anal. Calcd for C_28_H_18_O_4_N_2 _-Zn(II): C, 66.54%; H, 3.59; N, 5.54%; Co, 11.66%. Found: C, 66.54%; H, 3.60%; N, 5.55%; Co, 11.68%.

### 2.5. Synthesis of Metal-Containing Epoxy Polymers

The epoxidation of metal complexes was carried out by the reaction of Schiff base metal complexes (ML) with epichlorohydrin [14]. A mixture of (0.01 mol) ML dissolved in 20 mL DMF and 10 mL of epichlorohydrin was refluxedin a three-round-bottom flaskin the presence of sodium hydroxide(10 mL of 2*N*) was added gradually for 4 h. The progress of the reaction was monitored by TLC technique, and epoxide value as the heat evolution was slowed; the solution was poured in to ice cooled ether. The resulting colour precipitate of metal-containing epoxy polymers was filtered, washed with water and methanol, respectively, and dried in vacuum oven at 100°C for 2 h.

### 2.6. Antibacterial Assessments

 The antibacterial activities of the chelated epoxy polymers were performed according to the National Committee for Clinical Laboratory Standards (NCCLS) to determine minimum inhibitory concentration (MIC) values [15].The microorganisms used in this study were *S. aureus*,*B. subtilis *(Gram-positive bacteria) and *E. coli*,*P. aeruginosa *(Gram-negative bacteria). The strains were all cultured on Tryptic Soy Agar (TSA) (Difco, USA) and Mueller-Hinton Broth (MHB) (Difco, USA), incubated aerobically at 35.5°C overnight. For the growth culture, one colony from culture on the TSA was inoculated into the MHB and incubated aerobically at 35.5°C for 24 hours. Then bacterial concentrations were determined by measuring optical density (OD) at *λ* = 600 nm at 0.2 (OD of 0.2 corresponded to a concentration of 10^8^ CFU/mL) with a spectrophotometer.

The MIC_90%_ of the-metal-containing epoxy polymers was determined by modification of the broth dilution method in 96-well microtiter plate. The growth of bacteria was determined at the difference in absorbance after 24 hours incubation at 35.5°C. The absorbance at 600 nm was then determined by using microplate reader. All experiments were performed in triplicates against each tested microorganisms. The lowest concentration which inhibited microbial growth was reported as MIC_90%_ whereas minimal bactericidal concentration (MBC) was defined as the lowest concentration of the compound to kill the microorganisms [16].

## 3. Result and Discussion

### 3.1. Synthesis of Metal Complexes

**Scheme 1:**
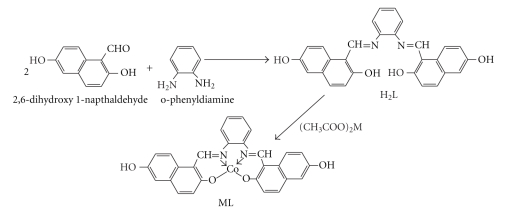


It has been known that Schiff base ligand was synthesized by the reaction of carbonyl compound and primary amine. In this study, we have synthesized H_2_L from 2,4 dihydroxy 1-napthaldehyde and o-phenylenediamine. The metal complexes were prepared by adding the methanolic solution of metal acetate to the THF solution of H_2_L in 1  :  1 molar ratio as given in Scheme 1. The synthesized metal complexes were soluble in DMSO, THF, and DMF but insoluble in methanol, ethanol, acetone, and water. The formation of H_2_L and its metal complexes was supported by elemental analysis, FTIR and ^1^HNMR spectroscopy. The FTIR spectrum of H_2_L showed a strong peak at 1641 cm^−1^, which was assigned to the C=N stretching in the case of metal complexes this peak was shifted to lower frequency at 1605–1610 cm^−1^, due to metal ions coordination through imines nitrogen. Two addition peaks at 620–662 cm^−1^ and 540–550 cm^−1^ were found in the spectra of metal complexes corresponding to M-O and M-N bond, respectively, [17].

The ^1^HNMR spectrum of H_2_L showed a resonance signal at 9.84 ppm for HC=N group, which had actually shifted downfield from its position in the spectrum of metal complexes and showed resonance at 9.16 ppm. The overall profiles of metal complexes are similar and supported by elemental analysis.

### 3.2. Synthesis of Metal-Containing Epoxy Polymers

**Scheme 2:**
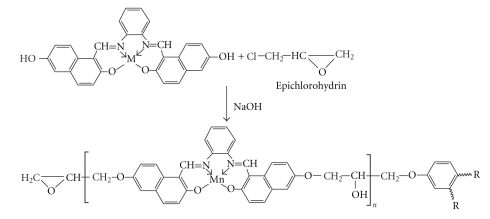


Epoxy resins are prepared by the step-polymerization of a bisphenol, and epichlorohydrin [18]. Herein, E-M(II) was prepared by the reaction of Schiff base metal complexes (LM) with epichlorohydrin in the presence of a sodium hydroxide according to Scheme 2. The reaction mechanism is similar to that we describe in our previous work [14].All of the synthesized metal-containing epoxy polymers were colored solids insoluble in water, ethanol, and methanol but soluble in DMF and DMSO. E-M(II) was prepared in a molar ratio of 2  :  1 epichlorohydrin to Schiff base metal complexes, which was supported by the physicochemical properties (Epoxy value, hydroxyl value, and Chlorine value) and elemental analysis, as listed in Table 1. The epoxy value of all the polymers was found in the range 0.18–0.22 mol/100 g. Medium molecular weights in the range 2225–2300 were found by the reduction of the amount of excess epichlorohydrin. The chlorine content of all the epoxy polymers was found to be 0.01–0.012 mol/100 g due to many side reactions such as dehydrohalogenation [19]. The secondary hydroxyl group content (hydroxyl value) was found in the range of 0.24–0.28 mol/100 g, which was formed along the chain molecule after the epoxy group was reduced. 

The spectra of all the chelated epoxy resin showed a broad band in the range 3345–3410 cm^−1^, assigned to *υ*(OH), which suggested the presence of hydroxyl groups. The presence of methylene groups in all the polymers was confirmed by the appearance of two strong bands at 2940 and 2860 cm^−1^ due to *υ*C-H symmetric and asymmetric stretching and a band at 1415 cm^−1^ due to the *δ*CH_2_ bending mode. All of the synthesized compounds showed additional absorption bands around 1260, 1165, and 890 cm^−1^ associated with epoxy groups although a band at 1260 cm^−1^ was identified with some reasonable certainty as being due to epoxy groups and a second band at 1155–1070 cm^−1^ was probably due to CH_2_-O vibrations when comparing their parental Schiff base ligand [20]. 

**Figure 1:**
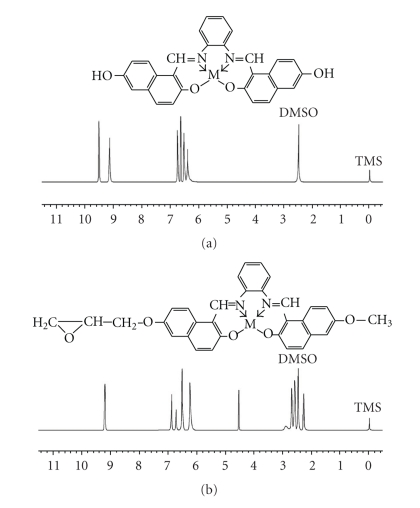
^1^H-NMR spectra of (a) (ZnL) Schiff base complexes and (b) Zn chelated epoxy polymers.

**Figure 2:**
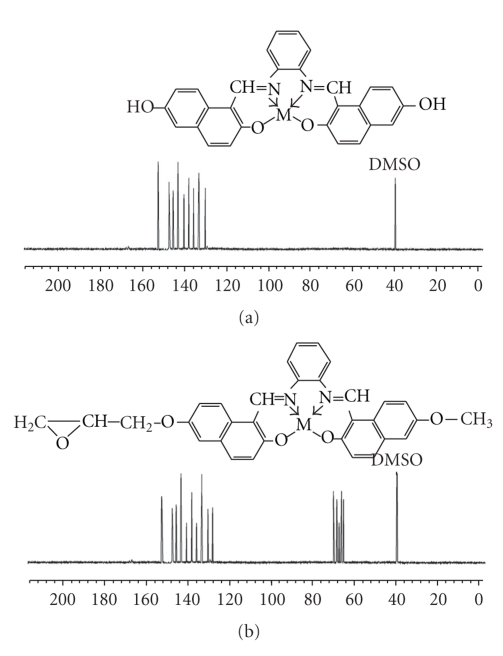
^13^C-NMR spectra of (a) (ZnL) Schiff base complexes and (b) Zn chelated epoxy polymers.

The ^1^H-NMR and ^13^C-NMR spectra of the diamagnetic metal-chelated epoxy resin were determined in DMSO-d_6_ and are given in Figures 1 and 2. The ^1^H-NMR spectra of these resins showed strong singlet signals at 9.20 ppm, which suggested azomethine protons (CH=N). The alcoholic protons (OH) showed a single resonance signal at 4.50 ppm in the case of E-Zn(II); this resonance signal was not found for ML. The chelated resin showed some other signals, assigned labels in Figure 1, at 2.20–3.02 ppm due to methylene protons in different environments. The number of protons calculated from the integration curves and those obtained from the values of the expected CHN analyses were in agreement. In the ^13^C-NMR spectra, Zn-chelated epoxy resin displayed signals assigned to CH=N carbons at 155 ppm. This signal appeared downfield in comparison with their original position (168 ppm), which indicated coordination with the central metal atom. A sharp peak at 62.2 ppm, assigned to the CH-OH function, was generated due to the reduction of oxirane groups with reactive hydrogen. Other resonance lines of these spectra fell into two main regions at 66.5–68.6 ppm for aliphatic carbons and 125–150.08 ppm for aromatic carbons [21]. 

**Figure 3:**
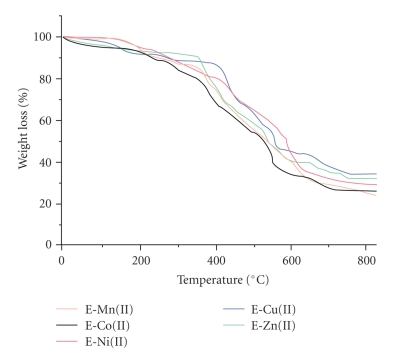
Thermogram of metal-chelated epoxy polymers.

A comparative study of the thermal behaviours of all the epoxy polymers was carried out in a nitrogen atmosphere with the purpose of examining the structure-property relationships at various temperatures, and results are given in Table 2. All of the polymers decomposed in two steps; the first step was faster than the second step as given in Figure 3. This may have been due to the fact that the non-coordinated part of the polymers decomposed first, and the actually coordinated part of the polymers decomposed later. The TGA trace of E-Cu(II) showed the initial decomposition at 450°C, about 10% weight loss was observed, which corresponded to an aliphatic portion/noncoordinated part such as CH_2_-CH-CH_2_ and epoxy groups per units of epoxy resin. Then, continued mass loss was observed up to 575°C, which indicated the decomposition and volatilization of the aromatic part into low-molecular-weight fractions, such as CH_4_, N_2_ and H_2_O. The thermogravimetric analysis (TG) of the chelated epoxy polymers revealed a mass loss in the temperature range 550–580°C, which corresponded to the formation of metal diisocyanate [M(OCN)_2_]. The next decomposition step occurred in the temperature range 610–800°C and corresponded to the thermal decomposition of M(OCN)_2_ to metal isocyanate [M(OCN)] and corresponded to the formation of MO [22]. The reduced masses of 34.20%, 32.05%, 28.50%, 25.21%, and 22.50% were found at 800°C, corresponded to E-Cu(II), E-Zn(II), E-Ni(II), E-Co(II) and E-Mn(II), respectively, and matched with Irvin-Williams order of stability of complexes of divalent metal ions. The observed reduced masses of all of the epoxy resin were greater than the calculated values; this was due to the formation of other compounds during the thermal reaction. Differential scanning calorimetry results of these epoxy resins revealed that the heat flow rate of the samples underwent a change during transition. The Tg values of all of the synthesized epoxy resins were computed from the results by the extrapolation of the pretransition and post transition line and by the calculation of the temperature when the heat flow rate was exactly in the middle of the pretransition and post transition rates. The Tg values of all of the synthesized polymers were in the range 180–220°C and are given in Table 2. All of the polymers showed a single Tg value due to the absence of any homopolymers, block polymers, and heterogeneous impurities [23].

### 3.3. Antibacterial Activity

The in vitro antibacterial activity of all the synthesized polymers was evaluated by using a minimum inhibitory concentration (MIC) and a minimum bactericidal concentration (MBC) procedure against *S. aureus*,*B. subtilis *(Gram-positive bacteria), and *E. coli,* *P. aeruginosa *(Gram-negative bacteria) bacteria accordance to methods of the National Committee for Clinical Laboratory Standards (NCCLSs) [15, 16]. The metal-chelated epoxy polymers inhibited growth of all the bacteria with MIC values ranging between 720 and 800 *μ*g/mL whereas MBC value was 850 *μ*g/mL displayed in Table 3. It was revealed that *E. coli* and *P. aeruginosa *were more sensitive to the chelated epoxy polymer than that of the other two bacteria due to the different component of bacterial cell wall. It was also observed that the E-Cu(II) showed higher MIC/MBC value 730/800, 740/810, 720/800 and 720/810 against *S. aureus*, *B. subtilis*,*E. coli,* and*P. aeruginosa *bacteria. On the other hand, the E-Mn(II) displayed lower antibacterial activity than that of other chelated epoxy polymers. In our previous study, we reported that chelation or coordination reduces the polarity of the metal ion by partial sharing of its positive charge with the donor groups and possibly p-electron delocalization within the whole chelate ring. This process thus increases the lipophilic nature of the compound, which, in turn, favors penetration through the bacterial wall of the microorganism. The Cu(II)-chelated resin showed the widest effective antibacterial due to a higher stability constant [24]. On the other hand, the difference in the magnitude of antimicrobial activity came from other factors, such as solubility, charge, and chirality of the polymers. Thus, it was apparent that the antibacterial activity of the chelated polymers of Schiff base was not only dependent on the charge but also on the chemical structure and nature of metal ions.

## 4. Conclusion

Metal-chelated epoxy polymers were successfully synthesized by the polycondensations of Schiff base metal complexes with epichlorohydrin in the basic medium and characterized by elemental, spectral, and thermal analysis. All the epoxy polymers in this study were colored solid, insoluble in common organic solvent but soluble in DMSO and DMF. The signal Tg value represents absence of any homopolymers, block polymers, and heterogeneous impurities. E-Cu(II) shows good thermal resistant as well as microbial resistant behaviour due to higher stability constant. Due to the promising thermal and microbial resistant behaviours, these polymers could be used as heat resistant-coating materials for aero space vehicles and antimicrobial coating materials in public palace and hospitals.

## Figures and Tables

**Table 1:** Physicochemical properties and elemental analysis of metal-cheated epoxy polymers.

Abbreviation	Yield (%)	Epoxy Value mol/100 g	Hydroxyl value mol/100 g	Chlorine value mol/100 g	Elemental Analysis (%)^a^			
					Carbon	Hydrogen	Nitrogen	metal
E-Mn(II)	70	0.22	0.24	0.010	66.56	4.27	4.57	8.95
					(66.54)	(4.26)	(4.58)	(8.97)
E-Co(II)	68	0.18	0.27	0.012	66.13	4.24	4.54	9.54
					(66.14)	(4.26)	(4.58)	(8.57)
E-Ni(II)	73	0.19	0.26	0.010	66.16	4.25	4.54	9.51
					(66.15)	(4.26)	(4.52)	(9.52)
E-Cu(II)	72	0.20	0.28	0.011	65.64	4.21	4.50	10.21
					(65.64)	(4.22)	(4.51)	(10.20)
E-Zn(II)	74	0.21	0.28	0.012	65.44	4.20	4.49	10.48
					(65.43)	(4.20)	(4.48)	(10.47)

^a^The values are presented as calculated (found).

**Table 2:** Thermal behaviors of metal-chelated epoxy polymers.

Abbreviation	T_g_ (°C)	Temperature (°C) corresponding to a weight loss					Char. (%) weight at 800°C
		10%	20%	30%	40%	50%	
E-Mn(II)	226	242	377	420	473	543	22.50
E-Co(II)	228	240	360	400	467	538	25.21
E-Ni(II)	231	277	400	460	537	596	28.50
E-Cu(II)	236	275	428	458	514	562	34.20
E-Zn(II)	234	360	382	422	498	544	32.05

Tg (°C)-glass transition temperature.

**Table 3:** Antibacterial activity of metal-chelated epoxy polymers.

Abbreviation	MIC/MBC (*μ*g/ML)^a^			
	*E. coli*	*P. aeruginosa*	*S. aureus*	*B. subtilis*
E-Mn(II)	800/850	790/825	760/840	780/840
E-Co(II)	800/840	750/840	750/830	740/830
E-Ni(II)	790/820	740/840	740/820	740/820
E-Cu(II)	730/800	730/810	720/800	720/810
E-Zn(II)	740/820	740/820	730/830	730/820

^a^MIC/MBC (±4), experiment were repeated three times.

## References

[1] Sridaeng D, Chantarasiri N (2009). Thermally stable polyureas and poly(urea-imide)s containing zinc and nickel napthtrien complexes. *Journal of Applied Polymer Science*.

[2] Cheng J-Y, Dong Y-B, Huang R-Q, Smith MD (2005). Synthesis and characterization of new coordination polymers generated from oxadiazole-containing ligands and IIB metal ions. *Inorganica Chimica Acta*.

[3] Kosobudskii ID, Kashkina LV, Gubin SP, Petrakovskii GA, Piskorskii VP, Svirskaya NM (1985). New type of metal-containing polymers- metallic clusters in polymer matrices. *Polymer Science U.S.S.R.*.

[4] Chantarasiri N, Damrongkosit T, Jangwong W, Sridaeng D, Suebphan S (2004). Synthesis, characterization and thermal properties of metal-containing polyurethane-ureas from hexadentate Schiff base metal complexes. *European Polymer Journal*.

[5] Chantarasiri N, Tuntulani T, Tongraung P, Seangprasertkit-Magee R, Wannarong W (2000). New metal-containing epoxy polymers from diglycidyl ether of bisphenol A and tetradentate Schiff base metal complexes. *European Polymer Journal*.

[6] Paul FB (1968). *Epoxy Resin Technology*.

[7] Chantarasiri N, Sutivisedsak N, Pouyuan C (2001). Thermally stable metal-containing epoxy polymers from an epoxy resin-Schiff base metal complex-maleic anhydride system. *European Polymer Journal*.

[8] Kurnoskin AV (1992). Heat resistance of metal-containing epoxy chelate polymers. *Polymer Degradation and Stability*.

[9] Cazacu M, Marcu M, Vlad A, Rusu GI, Avadanei M (2004). Chelate polymers. VI. New copolymers of the some siloxane containing bis(2,4-dihydroxybenzaldehyd-imine)Me^2+^ with bis(*p*-carboxyphenyl)diphenylsilane. *Journal of Organometallic Chemistry*.

[10] Matsuda H (1997). Polymers based on divalent metal salts of p-aminobenzoic acid: a review. *Polymers for Advanced Technologies*.

[11] Houjou H, Motoyama T, Araki K (2009). Electronic spectra of mono- and dinuclear complexes of fully *π*-conjugated salphen ligands synthesized by using 2,6-dihydroxynaphthalene carbaldehydes. *European Journal of Inorganic Chemistry*.

[12] Ekberov O, Basan S (1995). *Polymer Chemistry Lab*.

[13] Lee H, Neville K (1972). *Handbook of Epoxy Resine*.

[14] Nishat N, Ahmad S, Ahamad T (2006). Synthesis, characterization, and antimicrobial studies of newly developed metal-chelated epoxy resins. *Journal of Applied Polymer Science*.

[15] National Committee for Clinical Laboratory Standards (2000). *Methods for Dilution Antimicrobial Susceptibility Tests for Bacteria that Grow Aerobically. Approved Standard*.

[16] National Committee for Clinical Laboratory Standards (2002). *Performance Standards for Antimicrobial Susceptibility Testing. 8th Informational Supplement*.

[17] Ahamad T, Nishat N, Parveen S (2008). Synthesis, characterization and anti-microbial studies of a newly developed polymeric Schiff base and its metal-polychelates. *Journal of Coordination Chemistry*.

[18] Ahamad T, Nishat N (2008). New antimicrobial epoxy-resin-bearing schiff-base metal complexes. *Journal of Applied Polymer Science*.

[19] Sahmetlioglu E, Mart H, Yuruk H, Surme Y (2006). Synthesis and characterization of oligosalicylaldehyde-based epoxy resins. *Chemical Papers*.

[20] Colthup NB, Daly LJ, Wiberly SE (1975). *Introduction to Infrared and Raman Spectroscopy*.

[21] Nishat N, Parveen S, Dhyani S, Asma, Ahamad T (2009). Synthesis, characterization, and thermal and antimicrobial studies of newly developed transition metal—polychelates derived from polymeric Schiff base. *Journal of Applied Polymer Science*.

[22] Ahamad T, Kumar V, Nishat N (2009). New class of anti-microbial agents: synthesis, characterization, and anti-microbial activities of metal chelated polyurea. *Journal of Biomedical Materials Research A*.

[23] Anand M, Srivastava AK (1993). Synthesis and characterization of epoxy resins containing transition metals. *Polymer*.

[24] Kumar V, Ahamad T, Nishat N (2009). Some *O*, *O*′, *O*″, *O*‴-di/tetra aryldithioimidophonate transition metal complexes derived from catechol and bisphenol-A as antibacterial and antifungal agents. *European Journal of Medicinal Chemistry*.

